# PIF1 family DNA helicases suppress R-loop mediated genome instability at tRNA genes

**DOI:** 10.1038/ncomms15025

**Published:** 2017-04-21

**Authors:** Phong Lan Thao Tran, Thomas J. Pohl, Chi-Fu Chen, Angela Chan, Sebastian Pott, Virginia A. Zakian

**Affiliations:** 1 Department of Molecular Biology, Lewis Thomas Laboratory, Princeton University, Princeton, New Jersey 08544, USA; 2 Department of Human Genetics, University of Chicago, 920 E 58th St, Chicago, Illinois 60637, USA

## Abstract

*Saccharomyces cerevisiae* encodes two Pif1 family DNA helicases, Pif1 and Rrm3. Rrm3 promotes DNA replication past stable protein complexes at tRNA genes (tDNAs). We identify a new role for the Pif1 helicase: promotion of replication and suppression of DNA damage at tDNAs. Pif1 binds multiple tDNAs, and this binding is higher in *rrm3*Δ cells. Accumulation of replication intermediates and DNA damage at tDNAs is higher in *pif1*Δ *rrm3*Δ than in *rrm3*Δ cells. DNA damage at tDNAs in the absence of these helicases is suppressed by destabilizing R-loops while Pif1 and Rrm3 binding to tDNAs is increased upon R-loop stabilization. We propose that Rrm3 and Pif1 promote genome stability at tDNAs by displacing the stable multi-protein transcription complex and by removing R-loops. Thus, we identify tDNAs as a new source of R-loop-mediated DNA damage. Given their large number and high transcription rate, tDNAs may be a potent source of genome instability.

## Abstract (web summary)

The budding yeast genome encodes two Pif1 family helicases, Pif1 and Rrm3, previously shown to have distinct functions in the maintenance of telomeres and other aspects of genome stability. Here the authors identify a role for Pif1 (and Rrm3) in promoting DNA replication and suppressing R-loop mediated DNA damage at tRNA genes.

During each S-phase, the replication machinery encounters multiple classes of naturally occurring structures that impede fork progression. These structures include stable protein complexes, highly transcribed genes and stable DNA secondary structures^1^. Paused replication forks are particularly susceptible to breakage owing to the single-stranded DNA at the fork. Repair of these double strand breaks (DSBs) can promote recombination between direct repeats or lead to complex genetic events (gross chromosomal rearrangements, GCRs), which occur in many human cancers^2^.

Pif1 family DNA helicases are present in virtually all eukaryotes. While most eukaryotes, including humans, encode a single Pif1 family DNA helicase, *Saccharomyces cerevisiae* encodes two, Rrm3 and Pif1 (ref. 3). Although Pif1 and Rrm3 often affect the same substrates, they do not necessarily act in the same way^3^; e.g., Pif1 inhibits telomerase, while Rrm3 promotes semiconservative replication of telomeric DNA. In addition to inhibiting telomerase, Pif1 (but not Rrm3) is required for stable maintenance of mitochondrial DNA, Okazaki fragment maturation and break induced replication^4^. Pif1 also promotes replication and suppresses DNA damage at sequences that can form G-quadruplex structures *in vitro*^4^. Rrm3 is best known for promoting fork progression at stable protein–DNA complexes^5^,^6^. Rrm3 moves with the replication fork^7^, while Pif1 is probably recruited to its sites of action^8^ (C.-F.C., S.P., T.J.P. and V.A.Z., manuscript in preparation). Understanding the mechanism(s) by which Pif1 family helicases affect genome integrity is relevant to human disease, as mutation of mammalian PIF1 family helicases is associated with increased risk of breast cancer^9^ and mitochondrial myopathies^10^.

From yeasts to humans, stable protein–DNA complexes and R-loops are natural replication impediments^11^. Both of these structures occur at tDNAs. In *S. cerevisiae*, when replication and transcription move in opposite directions through a tDNA, forks slow even in wild type (WT) cells^12^. Rrm3 promotes fork progression at RNA Polymerase (Pol) III transcribed genes, such that in its absence, these genes become among the most potent naturally occurring impediments to fork progression^5^,^13^. Mutation of the tDNA promoter so that it is unable to assemble the transcription initiation complex eliminates pausing at the tDNA in both WT and *rrm3*Δ cells^5^. However, a more than 3-fold increase in the size of the transcribed tDNA does not increase the size or extent of replication pausing at the gene. These data suggest that Rrm3 acts by promoting fork movement past the particularly stable, multiprotein pre-initiation complex that is rapidly recycled such that many tDNAs are almost always pre-initiation complex associated 14,15. Using *S. cerevisiae* and genome-wide approaches, several laboratories have detected R-loops not only at highly transcribed RNA Pol II genes, but also at tDNAs^16^,^17^,^18^. From yeasts to humans, R-loops cause DNA damage at RNA Pol II transcribed genes^19^,^20^,^21^,^22^, but, to our knowledge, R-loop-mediated DNA damage has not been detected at RNA Pol III-transcribed genes in any organism.

There are ∼1,400 discrete sites in the *S. cerevisiae* genome that depend on Rrm3 for timely replication, including ∼425 RNA Pol III transcribed genes, such as the 274 tDNAs, ∼150 5S rRNA genes and other small RNAs (e.g., *SCR1* and *RPR1*)^5^,^13^,^23^. Although *rrm3*Δ cells are viable, they require DNA damage checkpoints and fork restart activities to survive^5^,^24^,^25^. Given the importance of Rrm3 for tDNA and 5S gene replication, we reasoned that there might be another DNA helicase that acts as a backup for Rrm3 at these hard-to-replicate sites. Indeed, *rrm3*Δ cells are not viable in the absence of at least two DNA helicases, Srs2 and Sgs1, but both of these helicases act after DNA replication, as the lethality in the double mutants (*srs2Δ rrm3*Δ and *sgs1*Δ *rrm3*Δ) is suppressed by deleting *RAD51* (refs 25,26). Although *pif1*Δ *rrm3*Δ cells are viable, they grow very slowly. Therefore, we hypothesized that Pif1 might act as a backup for Rrm3 during replication of tDNAs.

Here we show that tDNAs not only slow DNA replication, they also cause DNA damage, and this damage is due to R-loops. Pif1 binds robustly to multiple tDNAs. Fork pausing and R-loop-mediated DNA damage are both exacerbated in *pif1-m2 rrm3*Δ or *pif1*Δ *rrm3*Δ cells, compared to single mutants (*pif1-m2, pif1*Δ or *rrm3*Δ). Thus, the Pif1 family DNA helicases, Rrm3 and Pif1, act together to promote replication and suppress R-loop mediated DNA damage at tDNAs.

## Results

### Pif1 and Rrm3 bind to multiple tDNAs

**Figure 1:**
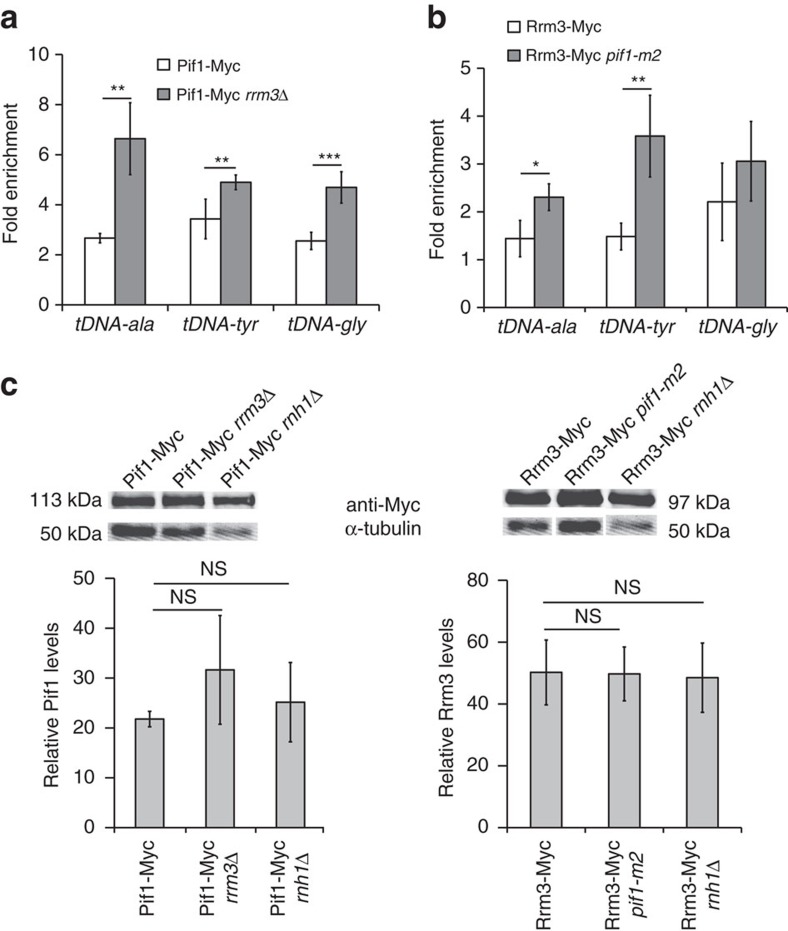
Pif1 and Rrm3 bind tDNAs. Enrichment of Pif1 (ChIP/input) or Rrm3 (ChIP/input) at tDNAs was normalized to helicase enrichment at *YBL028C*, a site that has low binding to both helicases. (**a**) Fold enrichment ([ChIP/input] _tDNA_/[ChIP/input] _*YBL028C*_) of Pif1-Myc in asynchronous wild-type (WT) and in *rrm3*Δ cells at *tDNA*^*ala*^HO, *tDNA*^*tyr*^HO and *tDNA*^*gly*^HO. (**b**) Fold enrichment of Rrm3-Myc in asynchronous WT and *pif1-m2* cells at *tDNA*^*ala*^HO, *tDNA*^*tyr*^HO and *tDNA*^*gly*^HO. Full blots are shown in Supplementary Fig. 7. (**c**) Western blot (top) and its quantification (bottom) showing Pif1 or Rrm3 expression levels in WT, *rrm3*Δ, *pif1-m2* and *rnh1*Δ cells. All quantification was normalized to the loading control, α-tubulin. The difference in Pif1 abundance between WT and *rrm3*Δ or *rnh1*Δ cells was not significant (NS) (*P*>0.05). Similarly, Rrm3 abundance was the same between WT and *pif1-m2* or *rnh1*Δ cells. Error bars are±s.d. for at least three independent experiments per strain. *P*-values here and elsewhere were obtained using unpaired two-tailed Student's *t*-test. Here and in subsequent figures, * indicates *P*<0.05, ** indicates *P*⩽0.009, and *** indicates *P*⩽0.0009.

**Figure 2:**
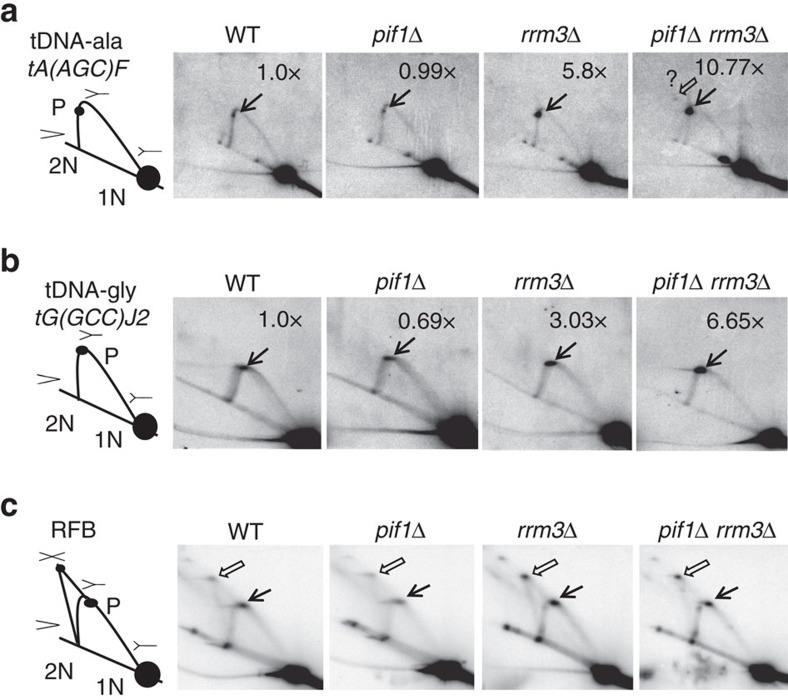
Forks stall but do not arrest at tDNAs in the absence of Pif1 family DNA helicases. DNA from asynchronous WT, *pif1*Δ, *rrm3*Δ or *pif1*Δ *rrm3*Δ cells was digested with restriction enzymes and analysed by two-dimensional (2D) gel electrophoresis and Southern hybridization. (**a**–**c**) Schematics of the results from 2D gel analysis for each region are shown to the left of the Southern blots of 2D gels: 1N marks the position of non-replicating linear fragments; 2N is position of almost fully replicated 1N fragments right before sister chromatids separate; P, replication pause; X, converged forks. Southern blots were hybridized using ^32^P-labelled probes to detect the following regions (see Supplementary Table 10 for primers for probes): (**a**) *tDNA*^*ala*^HO (BglII), (**b**) *tDNA*^*gly*^HO (EcoRI) and (**c**) rDNA (BglII fragment containing RFB). Black arrows indicate sites of fork pausing and open arrows indicate sites of converged forks. In **a**, there is a faint signal at tDNA^ala^ in *pif1*Δ *rrm3*Δ cells that might be due to converged forks; this signal is marked with an open arrow and a question mark. In **a**,**b**, the signal at the pause was quantified (see Supplementary Fig. 1 for details) and normalized to the pause signal in WT cells to obtain the relative fold change (top right corner of each Southern blot). The average fold difference of 2D gels from two independent biological replicates for *tDNA*^*ala*^ is shown in Supplementary Fig. 1. **c** is a 2D gel from the same DNA preparation used in **b** that was hybridized with an rDNA probe to visualize forks converged at the RFB.

As a first step to determine if Pif1 acts at tDNAs, we used chromatin-immunoprecipitation and quantitative PCR (ChIP-qPCR) to ask if Pif1 bound three tDNAs whose replication is Rrm3-sensitive^5^ (see also Fig. 2a,b and Supplementary Fig. 2): *tDNA*^*ala*^ (*tA(AGC)F*), *tDNA*^*tyr*^ (*tY(GUA)F1*) and *tDNA*^*gly*^ (*tG(GCC)J2*). For both Pif1 and Rrm3, their binding to tDNAs (ChIP/input) was normalized to *YBL028C*, a control sequence with low binding to both helicases. Pif1 binding to the three tDNAs was highly significant (P⩽0.0009) compared to the control sequence (Fig. 1a). Moreover, the binding of Pif1 to the three tDNAs was significantly higher in *rrm3*Δ than in WT cells (P⩽0.007). This increased Pif1 binding in *rrm3*Δ cells was not due to increased Pif1 abundance (Fig. 1c).

Because Rrm3 moves with the replisome, all nuclear sequences are Rrm3-associated at their time of replication^7^. Thus, it was not unexpected that Rrm3 binding to the three tDNAs was only modestly (1.5- to 2-fold) higher than to the control sequence (Fig. 1b). However, in *pif1-m2* cells, which are deficient in nuclear but not mitochondrial Pif1 (ref. 27), Rrm3 binding to two of the three tDNAs was significantly higher than in WT cells (*P*⩽0.01). This higher binding was not due to increased Rrm3 abundance in *pif1-m2* cells (Fig. 1c). Together, these binding patterns suggest that Pif1, like Rrm3, acts at tDNAs and that the action of both helicases at tDNAs may be more important in the absence of the other helicase.

### Pif1 is important for tDNA replication in *rrm3*Δ cells

The extent to which a tDNA impedes fork progression is dependent on the direction of replication through the gene. When replication and transcription move through a tDNA in the same direction (CD, co-directional), the tDNA has less impact on fork progression than when replication and transcription move in opposite directions (HO, head on orientation)^5^,^12^. The three tDNAs examined by ChIP-qPCR for Pif1 and Rrm3 binding (Fig. 1a,b) are all in the HO orientation^5^.

In previous work, we used two-dimensional gel (2D gels) electrophoresis^5^ and genome-wide analyses^13^ to show that fork progression is slowed at many, and perhaps all, tDNAs in the absence of Rrm3, and this effect occurred for tDNAs in both HO and CD orientations. Here we used 2D gels to determine if Pif1 affects replication at the three tDNAs analysed by ChIP-qPCR (Fig. 1a,b). Replication of each tDNA was examined at the gene's native chromosomal locus (Fig. 2a,b and Supplementary Fig. 1, 2). As expected, pausing at tDNAs was increased in *rrm3*Δ cells compared to WT cells (∼6-fold at *tDNA*^*ala*^HO and ∼3-fold at *tDNA*^*gly*^HO). The extent of pausing at both tDNAs was similar in *pif1*Δ and WT cells. However, in *pif1*Δ *rrm3*Δ cells, pausing at both tDNAs was significantly higher (∼11-fold at *tDNA*^*ala*^HO and ∼7-fold at *tDNA*^*gly*^HO) than in WT, *pif1*Δ or *rrm3*Δ cells. Similar results were seen for *tDNA*^*tyr*^HO (Supplementary Fig. 2). The replication pattern was comparable for *tDNA*^*ala*^HO when the partial loss of function allele *pif1-m2* was used in place of *pif1*Δ (Supplementary Fig. 4b). These data demonstrate that Pif1 promotes replication through tDNAs in *rrm3*Δ cells.

Recently, it was argued that forks do not pause but rather terminally arrest at tDNAs in *rrm3*Δ cells, and this terminal arrest is increased in cells lacking both Pif1 family helicases^28^. Terminal replication arrest generates converged replication forks at the site of the arrest, as seen by 2D gels at the replication fork barrier (RFB) in ribosomal DNA (rDNA)^29^. However, we saw no evidence for the X structures in *rrm3*Δ cells that are characteristic of converged forks at any of the eight tDNAs examined either here or in published 2D gels^12^ (see also Fig. 2a,b and Supplementary Figs 2c, 3, 4b).

One explanation for the failure to detect converged forks at tDNAs is that these structures were lost during DNA extraction or 2D gel analysis. To test this possibility, the same DNA preparation used to examine replication of *tDNA*^*gly*^HO (Fig. 2b) was tested subsequently for 2D gel analysis of rDNA replication (Fig. 2c). We chose an exposure where the signal in the 1N spot of unreplicated DNA molecules was lower in rDNA than in tDNA to ensure that the exposure for the tDNA was sufficient to detect converged forks at a single copy gene. The well-documented arrest and converged forks at the RFB were readily detectable in mutant and WT strains. Likewise, reprobing the Southern blots of *tDNA*^*ala*^HO 2D gels with the rDNA probe detected converged forks at the RFB (Supplementary Fig. 3).

Of course, even without terminal fork arrest, converged forks could occur transiently at tDNAs if moving forks from flanking origins meet at the tDNA. Fork convergence is more likely if the tDNA is about equi-distant from the two flanking origins. Convergence of moving forks at *tDNA*^*gly*^ is unlikely as this gene is predominately replicated by *ARS1018*, an early, efficient origin that is only 8.6 kb from the gene on its telomere-proximal side^30^,^31^,^32^. The nearest origin to *tDNA*^*gly*^ on the centromere-proximal side is *ARS1017,* an early origin that is located 72 kb away. However, at *tDNA*^*ala*^, the gene is 5.4 and 11.4 kb from the flanking origins (*ARS607* and *ARS608*, respectively)^31^,^32^,^33^. Thus, forks could converge occasionally at *tDNA*^*ala*^ if the more distal origin fires before the closer origin in some S phases. Indeed, while we saw no converged forks at *tDNA*^*ala*^ in WT, *pif1*Δ, or *rrm3*Δ cells (Fig. 2a; Supplementary Fig. 3), in *pif1*Δ *rrm3*Δ cells, there was an extremely weak but reproducible signal in the 2D gel that could be explained by forks converged at the tDNA (marked by open arrow and a question mark in Fig. 2a and Supplementary Fig. 3). We conclude from analysing replication of multiple tDNAs in their native chromosomal loci by 2D gels that fork arrest at tDNAs is very rare, not only in WT cells but also in the absence of Pif1 and/or Rrm3. Fork arrest at tDNAs is much rarer than in rDNA, where it is estimated to occur in fewer than 20% of the repeats^29^.

### Pif1 and Rrm3 suppress direct repeat recombination at tDNAs

**Figure 3:**
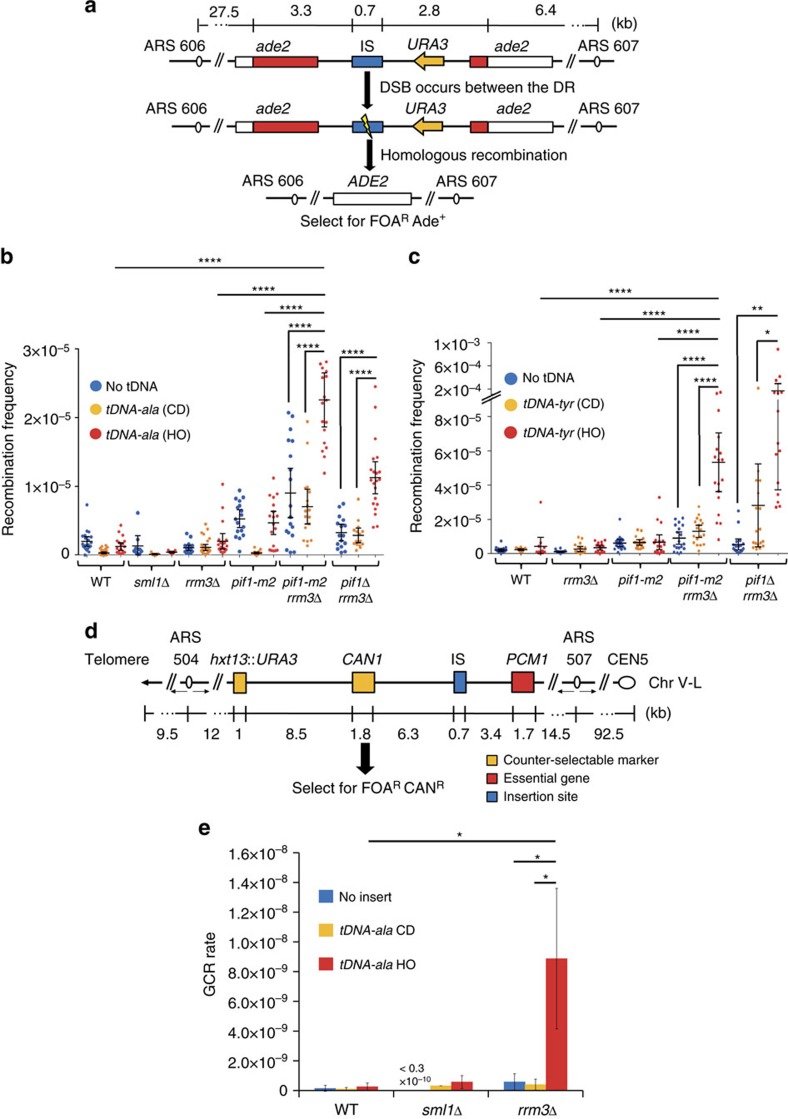
Pif1 family DNA helicases suppress DSB-induced recombination at tDNAs. (**a**) Schematic of the direct repeat (DR) recombination construct generated on the right arm of chromosome VI. Blue box indicates site of tDNA insertions (IS) where *tDNA*^*ala*^ or *tDNA*^*tyr*^ was inserted in head-on (HO) or co-directional (CD) orientation. Different parts of *ADE2* (red and white bars), neither of which is sufficient to generate Ade^+^ cells, are separated by ∼5 kb containing *URA3.* A double-strand break (DSB) between the *ade2* segments initiates recombination between the two partial copies of *ADE2*, which generates FOA^R^ Ade^+^ cells. (**b**,**c**) Recombination frequencies in cells with no insert or containing *tDNA*^*ala*^ (**b**) or *tDNA*^*tyr*^ (**c**) in HO or CD orientations were determined by dividing the number of FOA^R^ Ade^+^ cells by the number of viable cells that grew on non-selective plates. The means of the recombination frequencies with 95% confidence interval were calculated from at least three independent technical and biological replicates per strain. The mean±s.d. is also provided in Supplementary Tables 5 and 7. (**d**) Schematic of the left arm of chromosome V (Chr V-L) in gross chromosomal rearrangement (GCR) strains. *tDNA*^*ala*^HO or *tDNA*^*ala*^CD was inserted at insertion site (IS in blue box) in GCR strains. Genes within the 45 kb test interval are non-essential; *PCM1* (red box) is an essential gene, the most distal essential gene on ChrV-L. A DSB that occurs upstream of the two marker genes, *URA3* and *CAN1* (yellow boxes), initiates GCR events, which are monitored by selecting for FOA^R^ Can^R^ cells. (**e**) GCR rates with or without *tDNA*^*ala*^ CD or HO inserts were calculated using FALCOR and MMS maximum likelihood method^64^. Means and standard deviations of GCR rates were obtained from at least three technical replicates of two different isolates per strain. Here, * indicates *P*<0.05, ** indicates *P*⩽0.009, and **** indicates *P*<0.0001.

To determine if fork pausing at tDNAs causes DNA damage, we used two different genetic assays where the read-out is sensitive to the occurrence of DSBs. The first assay monitors recombination between direct repeats (DR) of portions of the *ADE2* gene, which are separated by a region containing *URA3.* In this assay, recombination generates FOA-resistant Ade2^+^ cells, and the occurrence of cells of this genotype is increased by a DSB between the repeats^8^,^34^ (Fig. 3a). By 2D gel analysis, *tDNA*^*ala*^HO caused fork pausing when inserted into the DR substrate, just as it did at its endogenous locus (Supplementary Fig. 4).

In both genetic assays, we determine if tDNAs affect recombination by comparing the number of recombination events within a strain with or without (‘no insert') a tDNA in the test interval. Insertion of *tDNA*^*ala*^HO (Fig. 3b) or *tDNA*^*tyr*^HO (Fig. 3c) in the DR assay did not cause a significant increase in recombination in *rrm3*Δ cells compared to the no insert control (Table 1). Similarly, in a *pif1-m2* strain, DR recombination was not increased significantly by the presence of either *tDNA*^*ala*^ or *tDNA*^*tyr*^ (CD and HO for both). However, the recombination frequency in *pif1-m2 rrm3*Δ cells was significantly higher in the presence of *tDNA*^*ala*^HO (∼2.5-fold) and *tDNA*^*tyr*^HO (∼6-fold) compared to no tDNA, *tDNA*^*ala*^CD or *tDNA*^*tyr*^CD (Table 1).

Because *pif1-m2* is not a null allele^27^, the DR recombination experiment was also carried out in *pif1*Δ and *pif1*Δ *rrm3*Δ cells (Fig. 3b,c and Supplementary Fig. 5b,c). Although mitochondrial deficiency increased the background level of DR recombination (Supplementary Fig. 5a), neither *tDNA*^*ala*^HO nor *tDNA*^*tyr*^HO affected the frequency of DR recombination in *pif1*Δ cells compared to the no insert control (Supplementary Fig. 5b,c). However, insertion of either tDNA caused a highly significant increase in DR recombination in *pif1*Δ *rrm3*Δ cells (3.6-fold for *tDNA*^*ala*^HO and 52.6-fold for *tDNA*^*tyr*^HO) (Fig. 3b,c and Table 1). This increase in *pif1*Δ *rrm3*Δ cells was higher than in *pif1-m2 rrm3*Δ cells. This result is consistent with *pif1-m2* being a partial loss of function allele. Taken together, these data indicate that Rrm3 and Pif1 act synergistically to suppress DNA damage, as inferred from the frequency of DR recombination at tDNAs.

### tDNAs increase gross chromosomal events in *rrm3*Δ cells

In *rrm3*Δ cells, DR recombination was increased at *tDNA*^*ala*^HO (1.9-fold) and *tDNA*^*tyr*^HO (3.2-fold), but neither increase was significant (Fig. 3b,c and Table 1). Because fork pausing increases dramatically at tDNAs in *rrm3*Δ cells^5^ (see also Fig. 2a,b), we decided to examine the effects of tDNAs on chromosome breakage in a GCR assay^35^, which is more sensitive than the DR assay. This assay detects complex genetic rearrangements within the non-essential ∼45 kb terminal portion of the left arm of chromosome V (Fig. 3d). (This assay is not well suited to study damage in *pif1-m2* or *pif1*Δ cells, as most DSBs in these backgrounds are repaired by telomere addition (TA), not recombination^36^; because *rrm3*Δ partially suppresses the TA phenotype of *pif1*Δ cells, GCR events in the double mutant arise by both TA and recombination^37^.)

As reported earlier^24^, in the parent strain (no tDNA in the test interval), *rrm3*Δ cells had a GCR rate similar to WT cells (Fig. 3e and Supplementary Table 1). In WT cells, insertion of *tDNA*^*ala*^HO or *tDNA*^*ala*^CD did not increase the GCR rate significantly (Fig. 3e and Table 2). In contrast, the GCR rate in *rrm3*Δ cells was 15-fold higher with *tDNA*^*ala*^HO than in the no insert control or in the *tDNA*^*ala*^CD. dNTP pools are increased in the absence of Rrm3 (ref. 38). To rule out the possibility that the increase of GCR rate in *rrm3*Δ cells was due to higher dNTP pools, GCR rates were also determined in a *sml1*Δ strain (*SML1* encodes an inhibitor of ribonucleotide reductase^39^). There was no increase in GCR rate in *sml1*Δ cells with *tDNA*^*ala*^HO or *tDNA*^*ala*^CD (Fig. 3e). Thus, the tDNA-dependent increase in GCR rate in *rrm3*Δ cells cannot be explained by increased dNTP pools.

### R-loops cause DNA damage at tDNAs

Genome-wide studies in *S. cerevisiae* detect R-loops at RNA Pol III transcribed genes, including tDNAs^16^,^17^,^18^. However, to our knowledge, the effects of R-loops at tDNAs on genome integrity have not been examined in any organism. Like R-loops at RNA Pol II transcribed genes^40^,^41^, R-loops at tDNAs are increased in RNaseH-depleted cells^16^,^17^,^18^. Thus, we determined if the tDNA-associated DNA damage seen in *rrm3*Δ and *pif1-m2 rrm3*Δ cells was due to R-loops. This hypothesis is appealing as Pif1 unwinds RNA-DNA hybrids *in vitro* much more efficiently than duplex DNA^42^,^43^.

First we asked if Pif1 and/or Rrm3 binding to the three tDNAs studied in other experiments in this paper was sensitive to the presence of R-loops by performing ChIP-qPCR in *rnh1*Δ cells, which lack RNaseH1. *S. cerevisiae* encodes a second RNaseH, Rnh201. Like Rnh1, Rnh201 removes R-loops, but it also removes ribonucleotides mis-incorporated during DNA replication^44^, and, therefore, its deletion increases genome instability^45^,^46^. As deleting *RNH1* does not cause genome instability^46^, we used Rnh1 deletion or Rnh1 overexpression for all R-loop assays. Because Rnh201 is present in *rnh1*Δ cells and able to destabilize R-loops, data in *rnh1*Δ cells underestimate the impact of R-loops on DNA damage at tDNAs.

**Figure 4:**
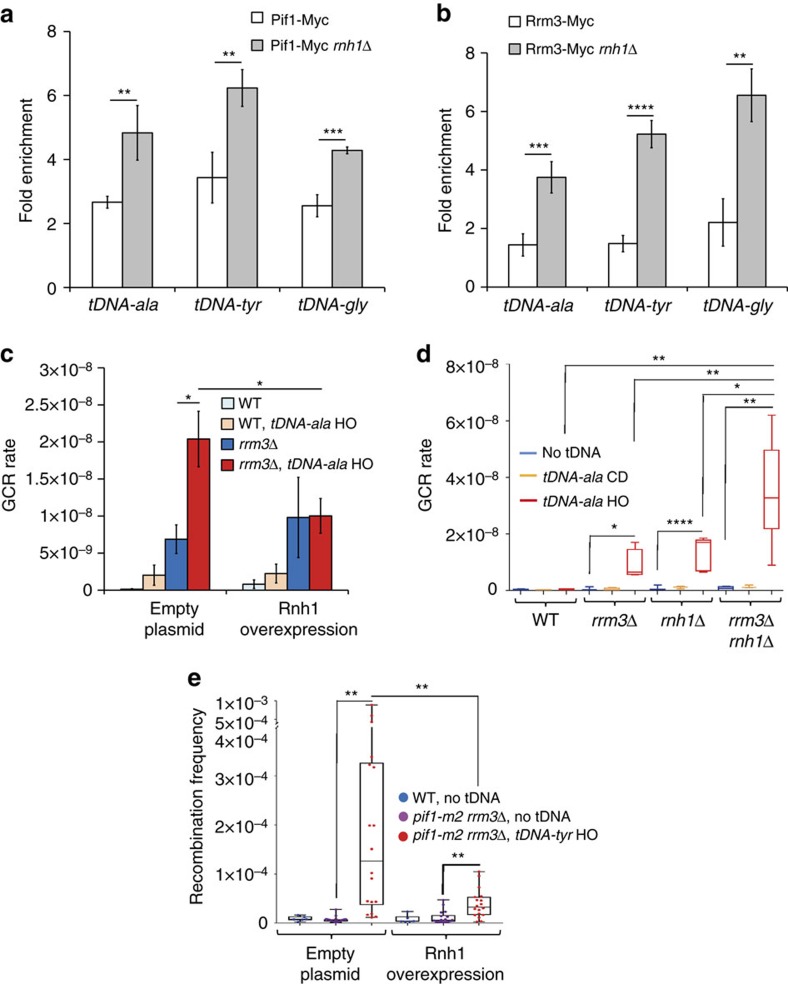
tDNA-mediated damage in cells lacking Pif1 family helicases is due to R-loops. In each panel, data are from at least three independent technical and biological replicates per strain. (**a**,**b**) ChIP-qPCR analyses of Pif1-Myc and Rrm3-Myc binding to *tDNA*^*ala*^HO, *tDNA*^*tyr*^HO and *tDNA*^*gly*^HO in asynchronous WT and *rnh1*Δ cells. Fold enrichment was calculated as in Fig. 1a,b. Error bars are ±s.d. for at least three independent experiments per strain. *P*-values here and elsewhere were obtained using unpaired two-tailed Student's *t*-test. (**c**) Means of GCR rates±s.d. in indicated strains. Cells carry either an empty or Rnh1 overexpression plasmid. (**d**) The distributions of events determined in the GCR assays in the indicated strains with or without *tDNA*^*ala*^ inserts are presented in box-and-whisker plots. The means of the GCR rates with 95% confidence interval were calculated from at least three independent technical and biological replicates per strain. Means of GCR rates±s.d. are also provided in Supplementary Table 1. (**e**) The recombination frequencies in the indicated strains with empty or Rnh1 overexpression plasmid are presented as box-and-whisker plots. The means of the recombination frequencies with 95% confidence interval were calculated from at least three independent technical and biological replicates per strain. Means of recombination frequencies±s.d are presented in Supplementary Table 7. Here, * indicates *P*<0.05, ** indicates *P*⩽0.009, *** indicates *P*⩽0.0009, and **** indicates *P*<0.0001.

Pif1 and Rrm3 association with all three tDNAs were significantly higher in the absence of Rnh1 (Fig. 4a,b). The abundance of Pif1 and Rrm3 was not affected by the absence of Rnh1 (Fig. 1c). These findings suggest that the functions of both Pif1 family helicases at tDNAs may be more important in cells with stabilized R-loops, as occurs in *rnh1*Δ cells.

To confirm the interpretation that tDNA-associated DNA damage in the GCR assay in *rrm3*Δ cells was due to R-loops, we used a plasmid containing a galactose-inducible *RNH1* gene^47^. The high GCR rate observed in *rrm3*Δ cells containing *tDNA*^*ala*^HO was significantly reduced by overexpression of Rnh1 (p<0.05) (Fig. 4c and Table 3). Indeed, when Rnh1 was overexpressed, the GCR rate was identical in *rrm3*Δ cells with and without *tDNA*^*ala*^HO. Likewise, deleting *RNH1*, which stabilizes R-loops^16^,^17^,^18^, significantly increased tDNA-mediated GCR events (*P*<0.0001) (Fig. 4d and Table 2).

We also determined the effects of Rnh1 overexpression on DR recombination in *pif1-m2 rrm3*Δ cells containing *tDNA*^*tyr*^HO (Fig. 4e and Table 3). As with the GCR rate in *rrm3*Δ cells, the frequency of DR recombination in *pif1-m2 rrm3*Δ cells was significantly reduced by Rnh1 overexpression (*P*=0.004) (Fig. 4e and Table 3). Taken together, these data make a compelling argument that DNA damage at tDNAs is due at least in part to R-loops.

## Discussion

Previous studies found that Pif1 and Rrm3 have non-overlapping functions at telomeres^37^, rDNA^23^, mitochondrial DNA^48^ and during break induced replication^49^. However, at G-quadruplex (G4) structures, the two helicases act similarly: Pif1 promotes replication and suppresses DNA damage at G4 forming sequences^8^,^50^,^51^,^52^, as does Rrm3 in cells lacking Pif1 (ref. 53).

Here we show that Pif1 is a backup for Rrm3 during replication of tDNAs. First, like Rrm3, Pif1 bound robustly to three of three tDNAs-HO, and this binding was higher in *rrm3*Δ cells (Fig. 1a). Second, by 2D gel analysis, pausing at the same three tDNAs was higher in *pif1*Δ *rrm3*Δ (or *pif1-m2 rrm3*Δ) than in *rrm3*Δ cells (Fig. 2a,b and Supplementary Fig. 2). Third, DNA damage was higher in *pif1*Δ *rrm3*Δ (or *pif1-m2 rrm3*Δ) cells than in single mutants at two of two tDNAs-HO (Fig. 3b,c).

Whereas 2D gels monitor fork progression, the genetic assays determine a possible downstream effect of pausing, DNA damage. Yeast tDNAs form R-loops^16^,^17^,^18^, and R-loops cause DNA damage at RNA Pol II genes^20^,^21^,^22^. To assess if R-loops cause damage at tDNAs, we determined the effects of different levels of RNaseH1 on tDNA-associated damage (Fig. 4c–e). In *rrm3*Δ cells, DNA damage at *tDNA*^*ala*^HO was reduced to the same level as at the ‘no insert' control by overexpressing Rnh1, which removes R-loops (Fig. 4c) and increased in *rnh1*Δ cells, which stabilizes R-loops (Fig. 4d). Likewise, the high level of DNA damage at *tDNA*^*tyr*^HO in *pif1-m2 rrm3*Δ cells was reduced by Rnh1 overexpression (Fig. 4e). We conclude that R-loops at *tDNA*^*ala*^HO and *tDNA*^*tyr*^HO are responsible, at least in part, for tDNA-induced DNA damage, and this damage is suppressed by Pif1 family helicases.

*In vitro* Pif1 has the unusual property of being particularly active at unwinding RNA–DNA hybrids^42^,^43^. It also displaces proteins from DNA^54^. Both of these activities are relevant to fork progression at tDNAs. Because Rrm3 is difficult to purify, its *in vitro* properties are not well characterized. However, its ability to suppress R-loop caused DNA damage at tDNAs (Fig. 4c,d) suggests that it is proficient at unwinding RNA–DNA hybrids *in vivo*. At several of its *in vivo* targets, such as tDNAs, the RFB, inactive origins and transcriptional silencers, Rrm3 promotes DNA replication by countering the inhibitory effects of stable protein complexes on fork progression^5^,^6^. Given its biochemical activities, Pif1 may back up Rrm3 not only by unwinding R-loops but also by displacing proteins, and these actions may occur both at tDNAs and other Rrm3 targets.

The effects of Pif1 family helicases on tDNA replication was recently examined genome-wide using sequencing of Okazaki fragments generated in DNA ligase I-deficient cells^28^. That study identified Pif1 is a backup for Rrm3 during tDNA replication. That study did not determine if tDNA pausing causes DNA damage, but observed that R-loops are not responsible for replication defects at tDNAs. In combination with our data, these results suggest that replication pausing and DNA damage at tDNAs are separable events.

While sharing some overlapping conclusions, our interpretations differ from those of Osmundson *et al*.^28^ regarding the nature of the replication defect at tDNAs. The patterns of Okazaki fragment abundance suggest that replication forks terminally arrest at tDNAs, leading the authors to argue that tDNAs are ‘point terminators'^28^. The authors sought to validate this interpretation using 2D gels. To this end, they examined replication of a single tDNA on a circular plasmid. Although structures consistent with converged forks at the tDNA are detected, the use of a circular plasmid makes it impossible to determine if they arise from pausing or arrest at the tDNA^28^. In contrast, we examined replication of multiple tDNAs at their native chromosomal locations and saw no convincing evidence for converged forks, even though converged forks in rDNA were readily detected in the same DNA samples (Fig. 2c and Supplementary Fig. 3). Likewise, in the original paper describing replication at tDNAs, the authors concluded that forks pause, rather than arrest, at tDNAs because they did not detect converged forks in their 2D gels^12^. The discrepancy in results between the two labs is probably due to the different methodologies. For example, analyses of the distribution of Okazaki fragments does not detect replication defects at several classes of well-documented pause sites, such as centromeres and highly transcribed RNA Pol II genes^28^. We conclude that forks pause, rather than arrest, at tDNAs, even in *pif1*Δ *rrm3*Δ cells where pausing was particularly robust. Of course, fork arrest may occur infrequently at many tDNAs, especially if the tDNA is similarly distant from flanking origins. Of the many tDNAs examined by 2D gels by us and others, the only hint of forks converging at a chromosomal tDNA gene was at *tDNA*^*ala*^, and even here the hint of converged forks was weak and only detected in *pif1*Δ *rrm3*Δ cells (Fig. 2 and Supplementary Fig. 3).

**Figure 5:**
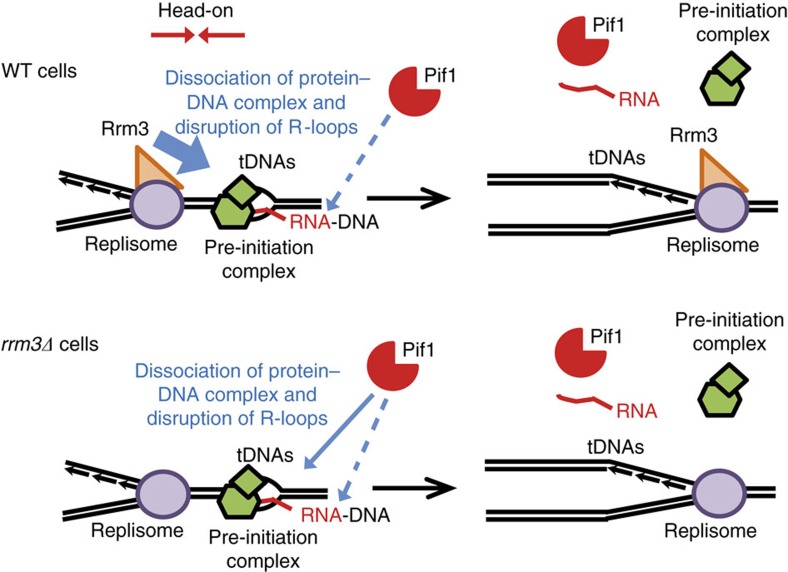
Model for Pif1 and Rrm3 function at tDNAs. During replication, Rrm3 plays the main role (thick blue arrow) in promoting replication fork progression and maintaining genome integrity at tDNAs by displacing both the RNA Pol III pre-initiation complex, which impedes fork progression, and R-loops, which cause DNA damage. Pif1 provides a backup for Rrm3 in both R-loop removal (dashed blue arrow) and protein displacement (thin blue arrow) at replicating tDNAs.

We propose a model where Rrm3 is usually responsible for dissociating the stable pre-initiation transcription complex and removing R-loops at tDNAs. Because Rrm3 moves with the replisome^7^, we propose that it acts at the time of tDNA replication (Fig. 5). Based on our ChIP, genetic and 2D gel data and Pif1's known biochemical activities, we propose that Pif1 has a back-up role in removing R-loops and proteins from tDNAs. Because fork pausing^5^,^12^ and DNA damage (Fig. 3b,c) were much higher at tDNAs-HO where transcription and replication collide than at tDNAs-CD, we think that most R-loop damage at tDNAs occurs during DNA replication (Fig. 5). However, Pif1, whose abundance is highest at the end of the S-phase^55^, may also promote R-loop and/or protein removal at tDNAs prior to chromosome condensation and mitosis.

In conclusion, our study links R-loops at tDNAs to DNA damage and uncovers roles for Pif1 family helicases in suppressing this damage. Pif1 family helicases are found in almost all eukaryotes and are highly conserved^3^. Pfh1, the fission yeast Pif1 family helicase, is critical for promoting fork progression and suppressing DNA damage at tDNAs, 5S genes and highly transcribed RNA Pol II genes^56^, and it unwinds RNA–DNA hybrids efficiently *in vitro* (M. Wallgren and N. Sabouri, personal communication). Hence, Pfh1 is also likely to suppress R-loop caused DNA damage. Eukaryotic genomes have a large number of highly transcribed RNA Pol III genes (e.g., ∼500 tDNAs and 400 5S rRNA genes in the haploid human genome)^57^,^58^. R-loops at RNA Pol III genes may be a heretofore unappreciated source of DNA damage that would occur in all cell types. There is increasing evidence that mutations in human genes that inhibit R-loop disassembly at RNA Pol II genes are associated with human disease^11^,^59^,^60^,^61^,^62^. If human PIF1, like its fungal relatives, resolves R-loops at RNA Pol III genes, this activity may be relevant to human health.

## Methods

### Yeast strains

All yeast strains were derivatives of YPH499 (ref. 63). Yeast strains, plasmids and primers are listed in Supplementary Tables 9–12. Gene disruptions and epitope tagging of proteins were confirmed by PCR, DNA sequencing, Southern blotting, western blotting and/or phenotypic analysis.

### Chromatin immunoprecipitation and quantitative PCR

Epitope tagging of proteins for ChIP experiments was done as previously described^7^,^8^. Briefly, cells were grown overnight and harvested at an OD_660_ of 0.5. Cells were crosslinked with 1% formaldehyde for 10 min. Chromatin purification was done as described^7^,^8^, except that DNA was sheared to an average size of 300 bps using E220 evolution Focused-ultrasonicator (Covaris, MA, USA). Anti-MYC monoclonal antibody (Clontech #631206) was diluted to 0.02 μg μl^−1^ and coupled to 80 μl of Dynabeads protein G (ThermoFisher #10004D). After crosslinking reversal and DNA purification, the immunoprecipitated and input DNA were analysed by qPCR using iQ SYBR Green Supermix (Bio-Rad #170-8882) and CFX96 real-time system (Bio-Rad). Samples were analysed in triplicates on three independent ChIP samples for each genotype. Strains and primers are listed in Supplementary Table 9. Wild-type YPH499 cells without an Myc-tagged protein were used as a control.

### Western blotting

Ten microlitres of cell extract from the input samples from ChIP experiments was analysed by western blotting. Briefly, cell extract was mixed with SDS–polyacrylamide gel electrophoresis sample buffer, boiled for 10 min and pelleted. Samples were loaded onto 15% (37.5:1 polyacrylamide:bis-acrylamide) SDS–polyacrylamide gel electrophoresis gels and run at 20 V cm^−1^. The proteins were transferred to a nitrocellulose membrane at 4 °C and blocked with 5% non-fat milk in TBST at room temperature using standard protocols. The blot was probed with an anti-MYC monoclonal antibody (Clontech #631206), which was diluted to 1:500 and visualized with an horseradish peroxidase -conjugated secondary antibody and ECL detection reagents (GE Healthcare). The loading control was obtained by reprobing the blot with an anti-alpha-tubulin antibody (Sigma #T6074) diluted 1:4,000. The quantification of protein levels is the mean of three different western blots. The full western blots are presented in Supplementary Fig. 7.

### DR recombination assay

DR recombination assays were done in yeast strain yBL3100, a derivative of YPH499 in which *ADE2* was integrated on chromosome VI between ORF *yFR020W* and *yFR021W.* Test sequences (*tDNA*^*ala*^ and *tDNA*^*tyr*^) from yeast DNA were cloned in both orientations into the pA2DRIV-B vector, which contains *URA3* and a portion of *ADE2* (ref. 34) (Fig. 3a). This vector with and without a tDNA was introduced by transformation into yBL3100 cells. The vectors were targeted into the inserted *ADE2* gene in a manner that disrupted *ADE2* to generate the DR substrate. Ura3^−^ Ade2^+^ recombinants were detected by growth on plates containing 5-FOA and lacking adenine. At least nine cultures from three independent replicates were grown to saturation in the absence of uracil for 2 days at 30 °C. Dilutions (1:10,000,000) were plated onto non-selective plates to determine the number of viable cells; 100–150 μl was plated on selective plates (1 g l^−1^ 5-FOA, no adenine). Plates were incubated for 4–5 days at 30 °C. Recombination frequencies were determined by dividing the number of recombinant cells by the number of viable cells. The mean recombination frequencies and standard deviations (s.d.) are from at least three independent technical and biological replicates per strain (Supplementary Table 5). *P*-values are calculated and presented in Supplementary Table 6.

To observe the effects of R-loops at tDNAs, DR strains were transformed with the p424 GAL1 vector or with the same vector containing Rnh1 expressed^47^ from the galactose-inducible GAL1 promoter (Supplementary Table 11, bottom). Two-millilitre cultures of each DR strain were grown overnight at 30 °C in medium containing 2% raffinose and no tryptophan (selects for plasmid) or uracil (selects against recombinants). Each culture were diluted to OD_660_ of ∼0.2 and grown in 2% galactose medium lacking both tryptophan and uracil for 24 h at 30 °C. Cells were pelleted and then resuspended to an OD_660_ of ∼0.25 in medium containing 2% galactose and no tryptophan and then grown for two generations at 30 °C. Dilutions were plated onto non-selective plates to determine the number of viable cells and selective plates (1 g l^−1^ 5-FOA, no adenine and no tryptophan) and grown for 4–5 days at 30 °C to determine the number of recombinants. Recombination frequencies were determined by dividing the number of recombinant cells by the number of viable cells. The means of recombination frequency and standard deviations were obtained from at least three independent technical and biological replicates per strain (Supplementary Table 7). *P*-values are calculated and presented in Supplementary Table 8.

### GCR assay

This GCR assay, which detects GCR events in the ∼45 terminal kb of the left arm of chromosome V, was developed in Richard Kolodner's lab^35^. GCR events are selected by the simultaneous loss of *URA3* and *CAN1* that are present naturally (*CAN1*) or by insertion (*URA3*) (Fig. 3d). The parent GCR strain was modified by inserting *tDNA*^*ala*^ between *PRB1* and *CIN8*. In a control experiment, we inserted *TRP1* instead of a tDNA into the test interval. Insertion of *TRP1* did not have a significant effect on GCR rates (Supplementary Fig. 8). To obtain GCR rates, sets of five 5-ml cultures of each *S. cerevisiae* GCR strain (Supplementary Table 11 top) were grown to saturation in YEPD medium at 30 °C for 2 days. Dilutions were plated onto non-selective plates and incubated at room temperature for 4 days to determine the number of viable cells. Cells were pelleted, resuspended in sterile water, plated on 5-FOA (1 g l^−1^) plus canavanine sulphate (60 mg l^−1^) minus uracil and arginine, and incubated at 30 °C for 4–5 days to determine the number of GCR events. GCR rates were calculated using FALCOR and the MMS maximum likelihood method^64^. Rates are the mean±s.d. of ≥3 independent experiments per strain (Supplementary Table 1). *P*-values are calculated and presented in Supplementary Table 2.

To observe the effects of RNA-DNA hybrids at tDNAs, GCR strains were transformed with an empty vector or the same vector expressing Rnh1 from the galactose inducible GAL1 promoter as described above (Supplementary Table 11 bottom). Sets of five 5 ml cultures of each GCR strain were grown to saturation at 30 °C for 4 days in medium containing 2% galactose and no tryptophan to maintain the vector. Each culture was plated on complete plates lacking tryptophan to determine the number of viable cells and on medium lacking tryptophan, uracil and arginine supplemented with 5-FOA (1 g l^−1^) and canavanine sulphate (60 mg l^−1^) to select for cells that lost expression of *URA3* and *CAN1*. The rates are the mean±s.d. of  ≥3 independent experiments per strain (Supplementary Table 3). *P*-values are calculated and presented in Supplementary Table 4.

### 2D agarose gel electrophoresis

Replication intermediates were analysed by standard 2D agarose gel electrophoresis techniques performed on total genomic DNA isolated from asynchronous cells^65^. Cells were collected in early log phase at OD_660_ of ∼0.6. Collected DNA was restriction enzyme digested (see figure legends for specific enzyme). In the first dimension, DNA was separated in 0.4% agarose at room temperature for 18–22 h depending on the size of the desired DNA fragment at 2.0 V cm^−1^. The second dimension was run for 13–15 h in 1.1% agarose containing ethidium bromide (0.3 μg ml^−1^) at 4.4 V cm^−1^ at 4 °C. Gels were analysed by Southern blotting using ^32^P-labelled probes. The extent of pausing was obtained using a typhoon scanner in the following manner. The ^32^P signal corresponding to the pause was obtained using ImageQuant TL software by first obtaining the overall intensity of the pause (Supplementary Fig. 1, label A^1^). Background signal was obtained by measuring intensity from an equal portion of the blot at a location that was offset from the pause site (Supplementary Fig. 1, label A^2^) and subtracted from the overall pause signal. The same steps were taken for a portion of the ascending y-arc (Supplementary Fig. 1, labels B^1^ and B^2^). The intensity of the pause for a given blot was normalized to replicating molecules by calculating the [pause/y-arc] ratio, [(A^1^-A^2^)/(B^1^-B^2^)]. Quantification of pausing was done in two independent biological replicates and was normalized to the WT pause signal to obtain fold increase relative to WT for each strain. This quantitation method is different from that used previously^5^, where extent of pausing was determined by dividing the ^32^P signal in the pause by the signal in the 1N spot using a Molecular Dynamics 400A PhosphorImager.

### Data availability

All data generated or analysed during this study are included in this published article and its Supplementary Files and are available from the corresponding author upon request.

## Additional information

**How to cite this article:** Tran, P. L. T. *et al*. PIF1 family DNA helicases suppress R-loop mediated genome instability at tRNA genes. *Nat. Commun.* **8,** 15025 doi: 10.1038/ncomms15025 (2017).

**Publisher's note:** Springer Nature remains neutral with regard to jurisdictional claims in published maps and institutional affiliations.

## Supplementary Material

Supplementary Information Supplementary Figures, Supplementary Tables and Supplementary References

## Figures and Tables

**Table 1:** Fold increase of DR recombination frequency relative to no tDNA insert.

**Strains**	**Fold increase of recombination frequency relative to no tDNA**			
	***tDNA***^***ala***^**CD**	***tDNA***^***ala***^**HO**	***tDNA***^***tyr***^**CD**	***tDNA***^***tyr***^**HO**
WT	0.2	0.6	1.2	2.1
*abf2*Δ	0.1	0.5	ND	ND
*pif1*Δ	0.6	0.5	1.0	1.3
*sml1*Δ	0.1	0.3	ND	ND
*rrm3*Δ	1.1	1.9	2.5	3.2
*pif1-m2*	0.05	0.8	1.1	1.1
*pif1-m2 rrm3*Δ	0.8	(*P*<0.0001) 2.5	1.4	(*P*<0.0001) 5.9
*pif1*Δ *rrm3*Δ	0.9	(*P*<0.0001) 3.6	8.9	(*P*=0.006) 52.6
ND, not determined.				

**Table 2:** Fold increase of GCR rate relative to no tDNA insert.

**Strains**	**Fold increase of GCR rate relative to no insert**	
	***tDNA***^***ala***^**CD**	***tDNA***^***ala***^**HO**
WT	0.7	1.8
*rrm3*Δ	0.7	(*P*=0.025) 15.3
*rrm3Δ rnh1*Δ	1.9	(*P*=0.005) 51.0

**Table 3:** Fold increase of GCR rate (*rrm3*Δ) or DR recombination frequency (*pif1-m2 rrm3*Δ) relative to no tDNA insert with or without Rnh1 overexpression.

**Strains**	**Fold increase relative to no insert**	
	**Empty plasmid**	**Rnh1** **overexpression**
*rrm3*Δ, *tDNA*^*ala*^HO	(*P*=0.017) 3.0	1.0
*pif1-m2 rrm3*Δ, *tDNA*^*tyr*^HO	(*P*=0.002) 29.9	(*P*=0.001) 3.5
