# Supplementary material for: PIF1 family DNA helicases suppress R-loop mediated genome instability at tRNA genes
Source: Nat Commun. 2017 Apr 21;8:15025. doi: 10.1038/ncomms15025 (PMC5413955; doi:10.1038/ncomms15025)
Supplement: Supplementary Information — Supplementary Figures, Supplementary Tables and Supplementary References [file ncomms15025-s1.pdf]

a

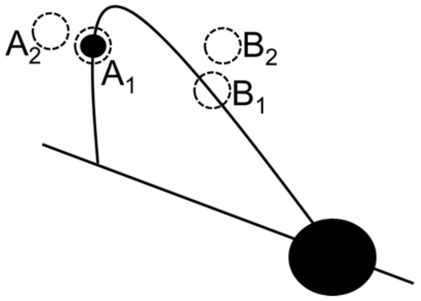

b

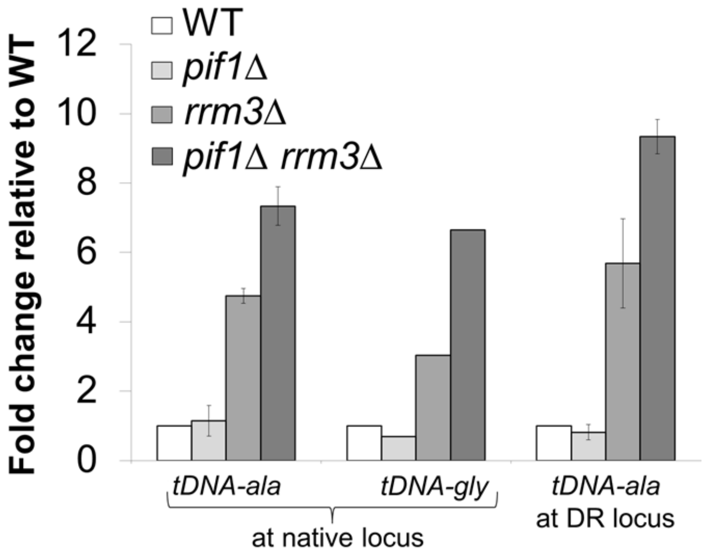

Supplementary Figure 1: (a) Schematic of 2D gel quantification. Pause signal for each strain was quantitated by measuring total pause signal (A1) relative to actively replicating molecules (B1) after removing background measured at A2 and B2, respectively, [(A1-A2)/(B1-B2)]. The fold change in pause intensity relative to WT was determined by [mutant pause/WT pause]. **(b) Fold increase of pausing relative to WT at endogenous *tDNA*^{ala}HO and *tDNA*^{gly}HO; and at *tDNA*^{ala}HO inserted at DR locus.** Quantification of pausing was normalized to WT to obtain fold increase relative to WT for each strain. *tDNA*^{gly} was done in one isolate. Error bars are ± standard error (s.e.) of two independent isolates for *tDNA*^{ala} at native and DR locus.

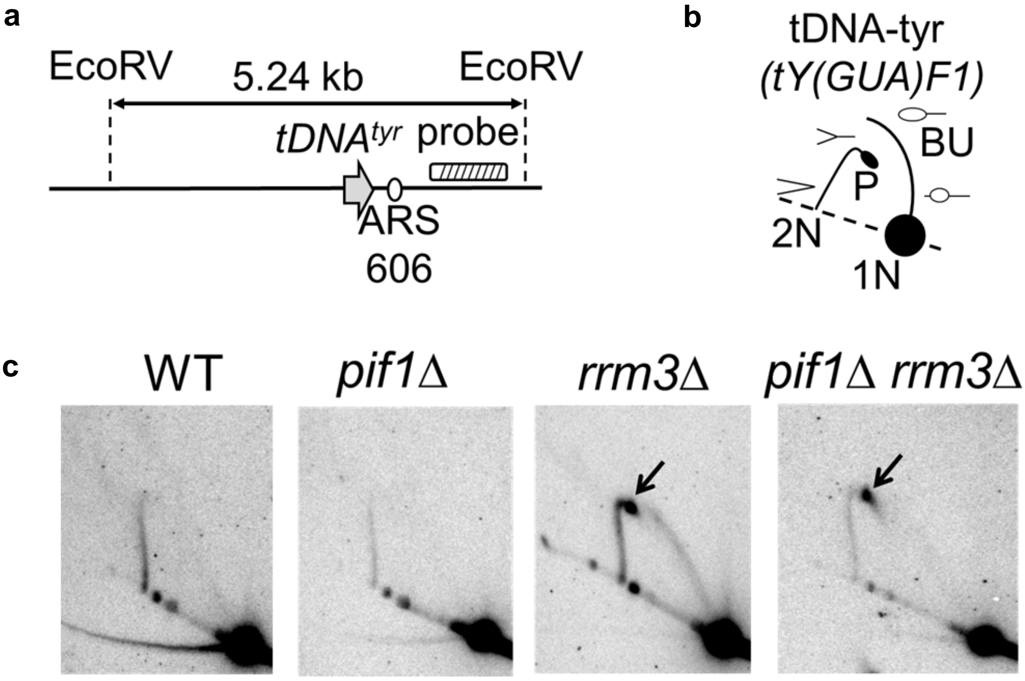

	[Pause / Ascending arc]
rrm3Δ	22.7
pif1Δ rrm3Δ	34.4

Supplementary Figure 2: (a) Map of $tDNA^{tyr}$HO at its endogenous locus. DNA from each strain was digested with EcoRV, which generates a 5.2 kb DNA fragment containing $tDNA^{tyr}$HO. Southern blots were probed using a ^{32}P labeled probe (dashed rectangle, see Supplementary Table 10 for primers). **(b) Schematic of $tDNA^{tyr}$HO 2D gel.** $tDNA^{tyr}$HO is located near *ARS606.* Therefore, in 2D gels of the EcoRV fragment, the pause (P) is located at the bubble (BU) to y-arc transition site. **(c) Southern blots of $tDNA^{tyr}$HO at its native locus in indicated strains.** In 2D gels of *rrm3*Δ and *pif1*Δ *rrm3*Δ cells, fork pausing is indicated by the solid arrow. Southern blots of WT and *pif1*Δ were too faint to see pausing. **(d) Quantification of fork pausing in $tDNA^{tyr}$*HO.*** Pausing in WT and *pif1*Δ cells was too faint to quantify. Therefore, pause intensity in *rrm3*Δ and *pif1*Δ *rrm3*Δ cells is displayed as the [Pause/Ascending arc] ratio. These data are consistent with those in Figure 2 and Supplementary Figure 4 where pausing is higher in *pif1*Δ *rrm3*Δ double mutant cells compared to *rrm3*Δ cells. Note that the exposure of the Southern for the double mutant is lower than that for the *rrm3*Δ DNA, as determined by intensity of the 1N spot. As a result, the pause at the tDNA in the double mutant appears lower than that for *rrm3*Δ.

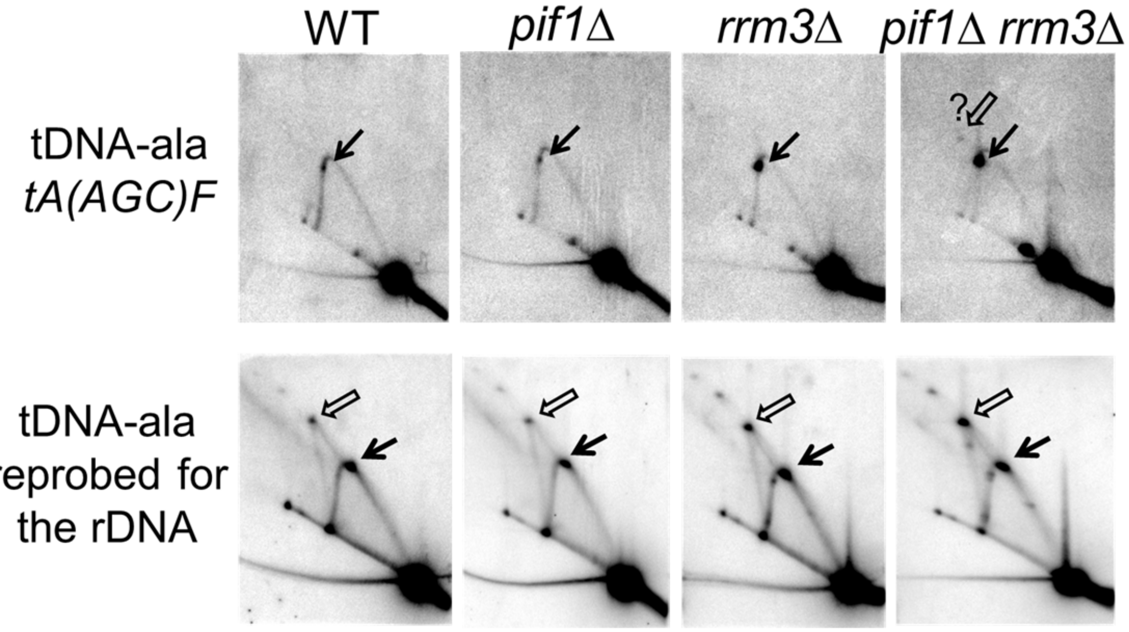

Supplementary Figure 3: Southern blots of *tDNA*[ala]HO at its native locus (top) is reprobed for the rDNA at RFB locus (bottom). DNA was digested with BglII and Southern blots were probed for the fragment containing *tDNA*[ala]HO (top panel) and for the fragment of the rDNA that contains the RFB (bottom). See Supplementary Table 10 for probe specific primers. In the top right panel, the open arrow and adjacent question mark are at a position consistent with forks converging at tDNA-ala.

a

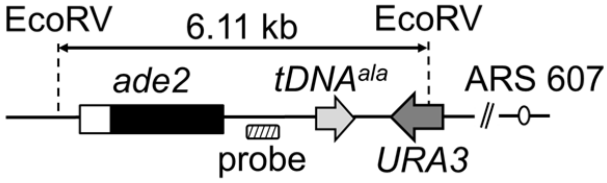

b

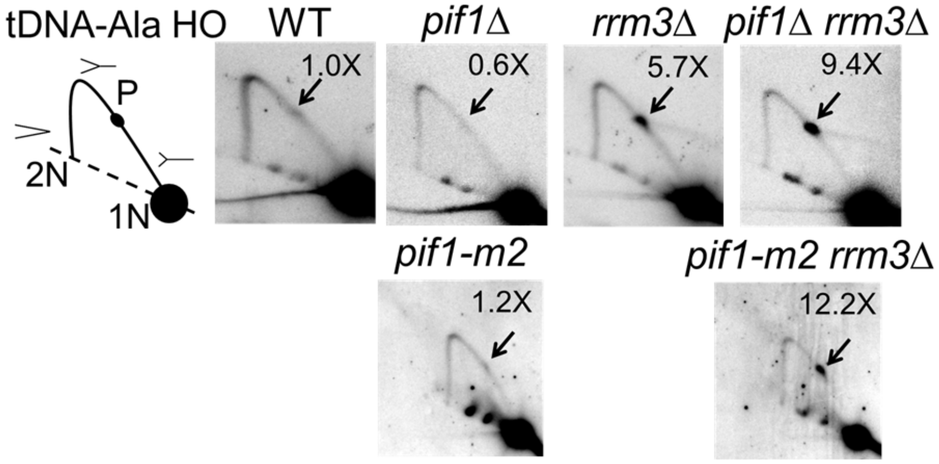

Supplementary Figure 4: (a) Map of *tDNA*^{ala}HO inserted at DR locus. DNA from each strain was digested with EcoRV, which generates a 6.1 kb DNA fragment containing the tDNA. Southern blots were probed using a ^{32}P labeled probe (dashed rectangle) that hybridizes to the bacterial ampicillin resistance gene that is part of the DR construct. See Supplementary Table 10 for primers. **(b) Southern blots of *tDNA*^{ala}HO inserted at DR locus in indicated strains.** 2D gels of a fragment containing *tDNA*^{ala}HO in the direct repeat context; also see quantification in Supplementary Figure 1. 1N, non-replicating fragment; 2N, almost fully replicated fragment right before sister chromatids separate; P, replication pause.

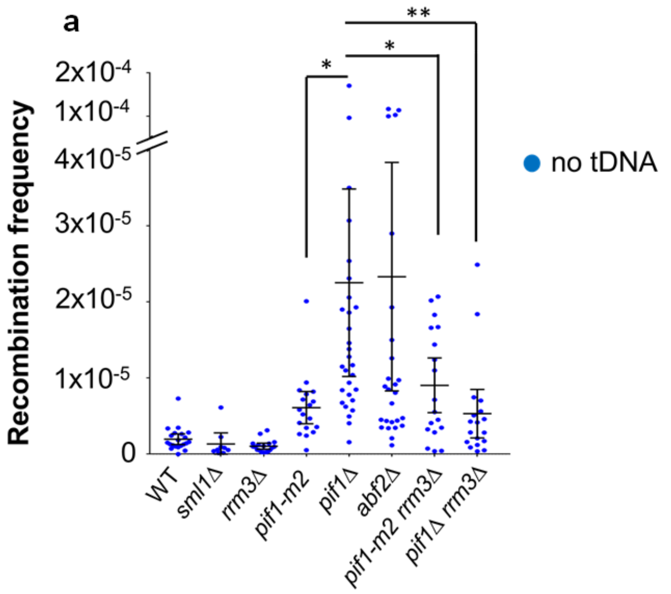

Fold increase of recombination frequency relative to WT	
**Strains**	**No insert**
abf2Δ	(p<0.0001) 11.9
pif1Δ	(p<0.0001) 11.5
sml1Δ	0.7
rrm3Δ	0.5
pif1-m2	(p=0.0008) 3.1
pif1-m2 rrm3Δ	(p=0.0007) 4.6
pif1Δ rrm3Δ	(p=0.043)1.6

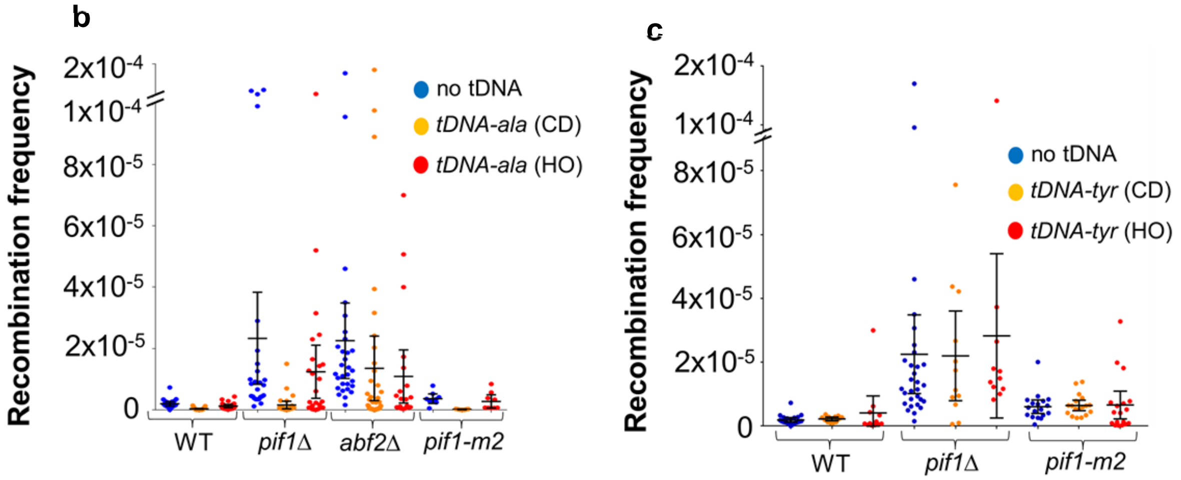

Supplementary Figure 5: (a) Recombination frequency of WT and mutants without insert (left) and table of fold increase in recombination relative to WT (right). *pif1Δ* cells exhibit a high and more variable recombination frequency that is likely due to defects in mitochondrial function, as it is also seen in mitochondrial deficient *abf2Δ* cells[1]. This interpretation is supported by the finding that these effects are not seen in *pif1-m2* cells, which are proficient in mitochondrial function[2] nor in *pif1Δ rrm3Δ* cells, where the respiratory defect of *pif1Δ* cells is largely suppressed[3,4] (see also Supplementary Figure 6). *p<0.5 and **p≤0.009. **(b, c) Recombination frequency of indicated strains in presence or absence of *tDNA*^{ala} or *tDNA*^{tyr} in HO or CD orientations.** There was no further increase in recombination in *pif1Δ, pif1-m2* or *abf2Δ* cells having *tDNA*^{ala}HO or *tDNA*^{tyr}HO in the test interval.

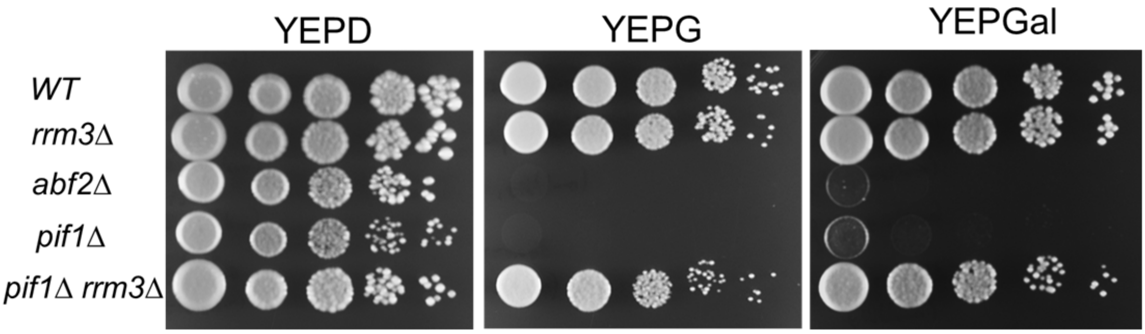

Supplementary Figure 6: Growth of the indicated strains on YEPD (D for dextrose), YEPG (G for glycerol) and YEPGal (Gal for galactose). Cells were grown overnight in liquid culture, diluted to OD_{660} = 1.4, and spotted onto YEPD, YEPG and YEPGal plates and grown for 3 days at 30°C. The experiment was done with three independent biological replicates for each genotype. Deletion of *ABF2* or *PIF1* results in a mitochondrial defect, causing death on YEPG and YEPGal. Deletion of *RRM3* in *pif1*Δ cells (*pif1*Δ *rrm3*Δ) partially rescues the mtDNA loss phenotype of *pif1*Δ cells, as described in[3,4].

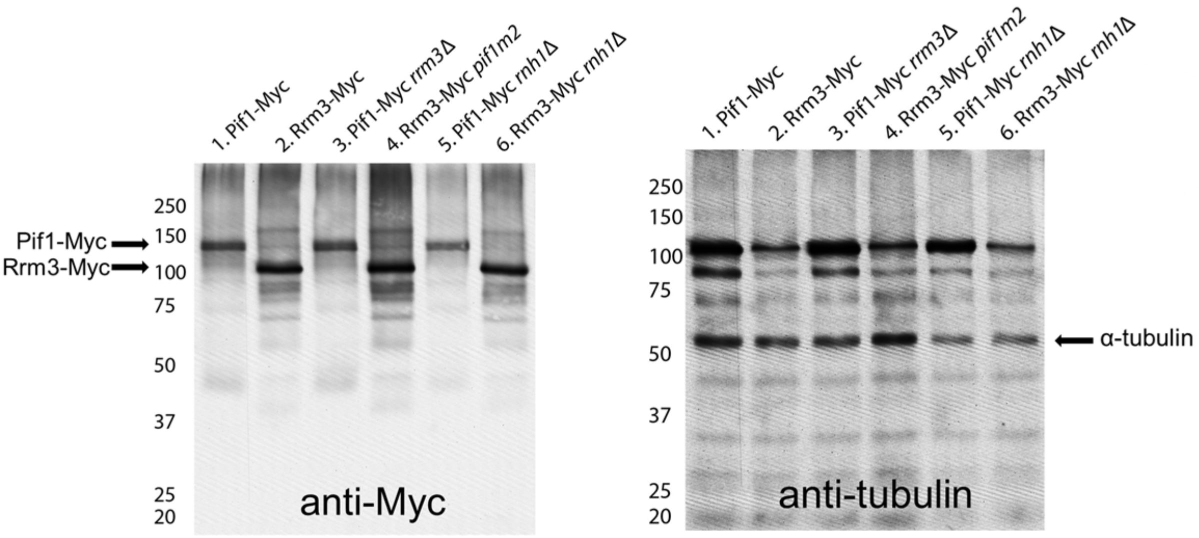

Supplementary Figure 7: Full western blot of Pif1-Myc (113kDa) and Rrm3-Myc (97kDa) expression levels in WT, *rrm3*Δ, *pif1-m2* and *rnh1*Δ cells (left). Full western-blot of α-tubulin (50kDa) expression level in WT and mutants (right).

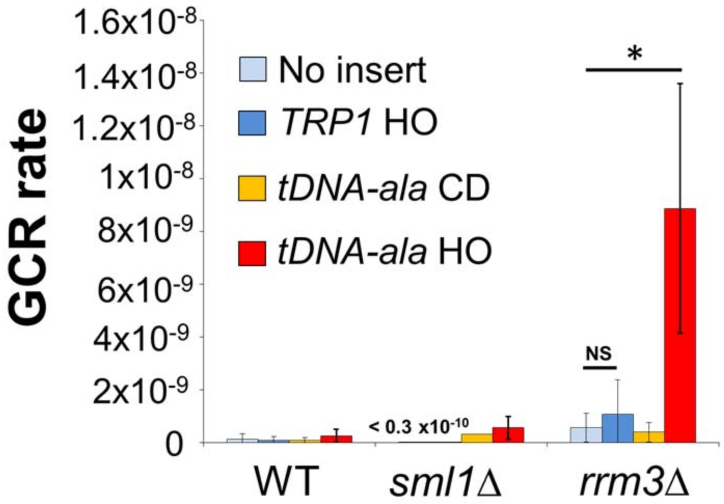

Supplementary Figure 8: GCR rates with or without *tDNA*^{ala} CD or HO inserts or with a transcribed non-tDNA, *TRP1* marker (in HO orientation) were calculated using FALCOR and MMS maximum likelihood method. Means and standard deviations of GCR rates were obtained from at least three technical replicates per strain. The data show no significant increase of GCR rate with *TRP1* (HO) inserted compared to no insert in WT, *sml1*Δ and *rrm3*Δ cells. Therefore, low level transcription as occurs at *TRP1* is not sufficient to increase the GCR rate significantly.

Supplementary Table 1: Mean GCR rate in indicated strains

Strains	Mean of GCR rate ($x10^{-10}$)							
	No insert		*TRP1*		*tDNA*^{ala}CD		*tDNA*^{ala}HO	
WT	1.46	±2.01	0.9	±1.5	1.02	±1.02	2.58	±2.50
*sml1*Δ	<0.3	ND	<0.2	ND	3.20	ND	5.73	±4.27
*rrm3*Δ	5.80	±5.45	10.8	±13	4.03	±3.65	88.70	±47.28
*rnh1*Δ	1.65	±7.60	ND	ND	10.16	±4.08	139.94	±47.79
*rrm3*Δ *rnh1*Δ	6.67	±6.67	ND	ND	12.37	±4.17	339.96	±163.32

Data are mean ± standard deviation (s.d.) calculated from ≥3 independent experiments. ND, not determined.

Supplementary Table 2: P values for data in Supplementary Table 1.

**P-value**	*rrm3*Δ, *tDNA*^{ala}HO
WT, *tDNA*^{ala}HO	P = 0.009**
*rrm3*Δ	P = 0.025*
*rrm3*Δ, *tDNA*^{ala}CD	P = 0.011*

**P-value**	*rrm3*Δ *rnh1*Δ, *tDNA*^{ala}HO
WT, *tDNA*^{ala}HO	P = 0.002**
*rrm3*Δ, *tDNA*^{ala}HO	P = 0.008**
*rnh1*Δ, *tDNA*^{ala}HO	P = 0.02*
*rrm3*Δ *rnh1*Δ, *tDNA*^{ala}CD	P = 0.002**

P-values are from ≥3 independent experiments and calculated using two-tailed unpaired student's t-test.

*P < 0.05 and **P ≤ 0.009

Supplementary Table 3: Mean GCR rate in indicated strains with or without Rnh1 overexpression

	Mean of GCR rate ($x10^{-10}$)			
**Strains**	empty plasmid		*Rnh1* overexpression	
WT	1.35	±0.65	8.09	±5.79
WT, *tDNA*^{ala}HO	20.23	±13.59	22.50	±12.70
*rrm3*Δ	68.80	±19.30	98.14	±54.14
*rrm3*Δ, *tDNA*^{ala}HO	203.93	±37.41	100.23	±23.41

Data are mean ± s.d. calculated from ≥3 independent experiments

Supplementary Table 4: P values for data in Supplementary Table 3

	*rrm3*Δ, *tDNA*^{ala}HO	
**P-value**	empty plasmid	*Rnh1* overexpression
WT	P = 0.001**	P = 0.058
*rrm3*Δ	P = 0.017*	P = 0.43

P-values are from ≥3 independent experiments and calculated using two-tailed unpaired student's t-test.

*P < 0.03 and **P ≤ 0.009

Supplementary Table 5: DR recombination frequency in indicated strains

Strains	Mean of recombination frequency (x10^{-6})									
	No insert		*tDNA^{ala}*CD		*tDNA^{ala}*HO		*tDNA^{tyr}*CD		*tDNA^{tyr}*HO	
WT	1.96	±1.54	0.31	±0.41	1.24	±1.17	2.27	±0.92	4.14	±8.36
abf2Δ	23.32	±37.2	2.04	±3.73	12.40	±21.9	ND	ND	ND	ND
pif1Δ	22.53	±33.0	13.5	±33.8	10.90	±19.0	22.03	±22.08	28.29	±38.29
sml1Δ	1.33	±1.91	0.10	±0.14	0.41	±0.19	ND	ND	ND	ND
rrm3Δ	1.05	±0.79	1.12	±1.19	2.00	±2.48	2.61	±2.95	3.41	±2.75
pif1-m2	6.10	±4.26	0.28	±0.29	4.66	±3.40	6.50	±3.30	6.632	±8.70
pif1-m2 rrm3Δ	9.03	±7.22	7.04	±4.91	22.5	±7.92	13.0	±7.19	53.35	±34.5
pif1Δ rrm3Δ	3.15	±2.68	2.85	±1.92	11.30	±5.13	28.14	±11.48	165.7	±59.94

Data are mean ± s.d. calculated from ≥3 independent experiments of 3 different biological isolates. ND stands for not determined.

Supplementary Table 6: P-values for data in Supplementary Table 5.

P-value	*pif1-m2 rrm3Δ, tDNA^{ala}*HO	*pif1-m2 rrm3Δ, tDNA^{tyr}*HO
WT, *tDNA$^{ala/tyr}$*HO	P < 0.0001****	P < 0.0001****
*rrm3Δ, tDNA$^{ala/tyr}$*HO	P < 0.0001****	P < 0.0001****
*pif1-m2, tDNA$^{ala/tyr}$*HO	P < 0.0001****	P < 0.0001****
pif1-m2 rrm3Δ	P < 0.0001****	P < 0.0001****
*pif1-m2 rrm3Δ, tDNA$^{ala/tyr}$*CD	P < 0.0001****	P = 0.0001***

P-value	*pif1Δ rrm3Δ, tDNA^{ala}*HO	*pif1Δ rrm3Δ, tDNA^{tyr}*HO
WT, *tDNA$^{ala/tyr}$*HO	P < 0.0001****	P = 0.024*
*rrm3Δ, tDNA$^{ala/tyr}$*HO	P < 0.0001****	P = 0.005**
*pif1-m2, tDNA$^{ala/tyr}$*HO	P < 0.0001****	P = 0.006**
pif1Δ rrm3Δ	P < 0.0001****	P = 0.006**
*pif1Δ rrm3Δ, tDNA$^{ala/tyr}$*CD	P < 0.0001****	P = 0.019*

P-values are from ≥3 independent experiments and calculated using two-tailed unpaired student's t-test.

*P < 0.05, **P ≤ 0.009, ***P ≤ 0.0009 and ****P < 0.0001

Supplementary Table 7: DR recombination frequency in indicated strains with or without Rnh1 overexpression

	**Mean of recombination frequency ($x10^{-6}$)**			
**Strains**	empty plasmid		*Rnh1* overexpression	
WT	8.75	±4.75	7.82	±8.11
pif1-m2 rrm3Δ	7.13	±5.97	11.23	±13.09
pif1-m2 rrm3Δ, *tDNA^{tyr}*HO	213.10	±117.37	39.03	±29.13

Data are mean ± standard deviation (s.d.) calculated from ≥3 independent experiments of 3 different biological isolates.

Supplementary Table 8: P-values for data in Supplementary Table 7

	pif1-m2 rrm3Δ, tDNA^{tyr}HO	
**P-value**	empty plasmid	*Rnh1* overexpression
WT	P = 0.002**	P = 0.0005***
pif1-m2 rrm3Δ	P = 0.002**	P = 0.001**

**P-value**	*pif1-m2 rrm3Δ, tDNA^{tyr}HO* empty plasmid
pif1-m2 rrm3Δ tDNA^{tyr}HO *Rnh1* overexpression	P = 0.002**

**P ≤ 0.009 and ***P ≤ 0.0009

Supplementary Table 9: Yeast strains and ChIP-qPCR primers

Strain	Genotype	Reference or source
YPH499	*MATa ura3–52 lys2–801 ade2–101 trp1-Δ63 his1-Δ200 leu2-Δ1*	Lab stock
YCF101	YPH499 *PIF1*-MYC13::*TRP1*	Paeschke et al.,Cell, 2011[5]
YCF103	YPH499 *RRM3*-MYC13::*TRP1*	Azvolinsky et al.,Gene Dev, 2006[6]
YCF104	YPH499 *PIF1*-MYC13::*TRP1* bar1Δ::*NAT*	This study
YCF105	YPH499 *RRM3*-MYC13::*TRP1* bar1Δ::*NAT*	This study
YCF106	YPH499 *PIF1*-MYC13::*TRP1* bar1Δ::*NAT* rrm3Δ::*HIS3*	This study
YCF107	YPH499 *RRM3*-MYC13::*TRP1* bar1Δ::*NAT* pif1-m2::*HPH*	This study
YCF108	YPH499 *PIF1*-MYC13::*TRP1* bar1Δ::*NAT* rnh1Δ::*HPH*	This study
YCF109	YPH499 *RRM3*-MYC13::*TRP1* bar1Δ::*NAT* rnh1Δ::*HPH*	This study

Primer name	Primer sequence
tDNA-ala - *tA(AGC)F* - ChVI, For	GGGAAAGATTGTACGGGAAATG
tDNA-ala - *tA(AGC)F* - ChVI, Rev	ACCTGTATCATTTCTGTTTGGAAG
tDNA-tyr - *tY(GUA)F1* - ChVI, For	CCTCTGCGTCTCATTGGAAGAAG
tDNA-tyr - *tY(GUA)F1* - ChVI, Rev	GACCAACCAAATCAATAATAAAATTCGGC
tDNA-gly – ChX, For	CTGTACACATAATTGACACGTTTAAC
tDNA-gly – ChX, Rev	ACTTCGTCCCAATCCTTTATTCC
YBL028C – ChII, For	TCTCGTCGACATCCATGTCAATTCC
YBL028C – ChII, Rev	GACACTGATGACAAACCAAGAGTTCA

Supplementary Table 10: Primers used for 2D gel probes

Primer name	tDNA	Primer sequence
DR 2D (tDNA_alanine in DR), For	tA(AGC)F	TGGCGTTTTTCCATAGGCT
DR 2D (tDNA_alanine in DR), Rev	tA(AGC)F	TGTCGCCCTTATTCCCTTTT
AGC/F (tDNA-ala native)	tA(AGC)F	TGCACTCACACCATTCACACT
AGC/R (tDNA-ala native)	tA(AGC)F	ATTGGCCCAAAAGGGATCAT
GUA/F (tDNA-tyr native)	tY(GUA)F1	CTTTTTCTGGCTTTATGATATGTTG
GUA/R (tDNA-tyr native)	tY(GUA)F1	ATTTGGTTGTTGTAGTTGATATTGG
tRNA (gly) tG(GCC)J2 F	tG(GCC)J2	AAACCGACCAAAAAGAGGTG
tRNA (gly) tG(GCC)J2 R	tG(GCC)J2	AACGTAAGTCCAGGCCTCATT
rDNA 2D F (RFB)		AGCCTGCTATGGTTCAGCGA
rDNA 2D R (RFB)		TTTATCCGGAGATGGGGTCTT

Supplementary Table 11: Yeast strains and plasmids used in GCR assays

Strain name	Gene(s) of interest	Insert	Reference or source
TTY 156	WT	no insert	This study
*TTY164 / 165	WT	*TRP1* HO	This study
TTY 170	WT	*tDNA*^{ala}CD	This study
TTY 171	WT	*tDNA*^{ala}HO	This study
*TTY 168 / 169	*sml1*Δ	no insert	This study
*TTY 188 /189	*sml1*Δ	*TRP1* HO	This study
*TTY 677 / 678	*sml1*Δ	*tDNA*^{ala}CD	This study
*TTY 196 / 197	*sml1*Δ	*tDNA*^{ala}HO	This study
*TTY 316 / 317	*rrm3*Δ	no insert	This study
*TTY 184 /185	*rrm3*Δ	*TRP1* HO	This study
*TTY 681 / 682	*rrm3*Δ	*tDNA*^{ala}CD	This study
*TTY 194 / 320 / 321	*rrm3*Δ	*tDNA*^{ala}HO	This study
TTY 331	*rnh1*Δ	no insert	This study
TTY 338 / 339	*rnh1*Δ	*tDNA*^{ala}CD	This study
*TTY 441 / 442 / 443	*rnh1*Δ	*tDNA*^{ala}HO	This study
*TTY 410 / 411	*rrm3*Δ *rnh1*Δ	no insert	This study
*TTY 444 / 445 / 446	*rrm3*Δ *rnh1*Δ	*tDNA*^{ala}CD	This study
*TTY 340 / 412 / 414	*rrm3*Δ *rnh1*Δ	*tDNA*^{ala}HO	This study

*Different biological isolates were tested for each genotype. All strains are in YPH background.

Plasmid name	Gene(s) of interest	Reference or source
TTB39 or pGALS-424	*GAL1*, *TRP1* (empty vector)	Lab stock
TTB40 or pGALS-424-*RNH1*	*GAL1, TRP1, RNH1* (RNH1 overexpression vector)	This study

Supplementary Table 12: Yeast strains and plasmids used in DR assay and 2D gels

Strain name	Gene(s) of interest	Insert	Reference or source
yBL3100	ChrVI-Ade^{+}	--	Brian Lenzmeier[7] (lab stock)
TTY 533 / 534 / 535	WT	no insert	This study
TTY 232 / 233 / 234	WT	$tDNA^{ala}$CD	This study
TTY 536 / 537 / 538	WT	$tDNA^{ala}$HO	This study
TTY 575 / 576 / 577	*abf2*Δ	no insert	This study
TTY 539 / 540 / 541	*abf2*Δ	$tDNA^{ala}$CD	This study
TTY 565 / 566 / 567	*abf2*Δ	$tDNA^{ala}$HO	This study
TTY 582 / 583 / 584	*pif1*Δ	no insert	This study
TTY 530 / 531 / 532	*pif1*Δ	$tDNA^{ala}$CD	This study
TTY 527 / 528 / 529	*pif1*Δ	$tDNA^{ala}$HO	This study
TTY 572 / 573 / 574	*sml1*Δ	no insert	This study
TTY 585 / 586 / 587	*sml1*Δ	$tDNA^{ala}$CD	This study
TTY 568 / 569 / 570 / 571	*sml1*Δ	$tDNA^{ala}$HO	This study
TTY 612 /613 / 614 / 635	*rrm3*Δ	no insert	This study
TTY 304 / 305 / 306 / 307	*rrm3*Δ	$tDNA^{ala}$CD	This study
TTY 556 / 557 / 558	*rrm3*Δ	$tDNA^{ala}$HO	This study
TTY 597 / 672 / 673 / 674	*pif1*Δ *rrm3*Δ	no insert	This study
TTY 598 / 599 / 600	*pif1*Δ *rrm3*Δ	$tDNA^{ala}$CD	This study
TTY 594 / 595 / 596	*pif1*Δ *rrm3*Δ	$tDNA^{ala}$HO	This study
TTY 715 / 716 / 717	*pif1-m2*	no insert	This study
TTY 718 / 719 / 720	*pif1-m2*	$tDNA^{ala}$CD	This study
TTY 722 / 723 / 724	*pif1-m2*	$tDNA^{ala}$HO	This study
TTY 736 / 737	*pif1-m2 rrm3*Δ	no insert	This study
TTY 739 / 740 / 741	*pif1-m2 rrm3*Δ	$tDNA^{ala}$CD	This study
TTY 743 / 744 / 745	*pif1-m2 rrm3*Δ	$tDNA^{ala}$HO	This study
yBL68	WT	$tDNA^{tyr}$CD	Brian Lenzmeier[7] (lab stock)
yBL136	WT	$tDNA^{tyr}$HO	
TTY 657 / 658 / 659 / 660	*pif1*Δ	$tDNA^{tyr}$CD	This study
TTY 661 / 662 / 663 / 664	*pif1*Δ	$tDNA^{tyr}$HO	This study
TTY 665 / 666 / 667	*rrm3*Δ	$tDNA^{tyr}$CD	This study
TTY 668 / 669 / 670	*rrm3*Δ	$tDNA^{tyr}$HO	This study
TTY 703 / 704 / 705	*pif1*Δ *rrm3*Δ	$tDNA^{tyr}$CD	This study
TTY 687 / 688 / 700 / 701	*pif1*Δ *rrm3*Δ	$tDNA^{tyr}$HO	This study
TTY 724 / 727 / 728	*pif1-m2*	$tDNA^{tyr}$CD	This study
TTY 730 / 731 / 732	*pif1-m2*	$tDNA^{tyr}$HO	This study
TTY 746 / 748 / 749	*pif1-m2 rrm3*Δ	$tDNA^{tyr}$CD	This study
TTY 750 / 751 / 752	*pif1-m2 rrm3*Δ	$tDNA^{tyr}$HO	This study

Three different biological isolates were tested for each genotype. All strains are in YPH background.

Plasmid name	Gene(s) of interest	Reference or source
pA2-DRIVB	*URA3,* partial *ade2*	Brian Lenzmeier[7] (lab stock)
TTB48	pA2-DRIVB, $tDNA^{ala}$CD	This study
TTB65	pA2-DRIVB, $tDNA^{ala}$HO	This study
TTB50	pA2-DRIVB without inserted region	This study

References for Supplementary Info

1. Cho, J.H., Ha, S.J., Kao, L.R., Megraw, T.L. & Chae, C.B. A novel DNA-binding protein bound to the mitochondrial inner membrane restores the null mutation of mitochondrial histone Abf2p in Saccharomyces cerevisiae. *Molecular and Cellular Biology* **18**, 5712-23 (1998).
2. Schulz, V.P. & Zakian, V.A. The saccharomyces PIF1 DNA helicase inhibits telomere elongation and de novo telomere formation. *Cell* **76**, 145-55 (1994).
3. Cheng, X., Qin, Y. & Ivessa, A.S. Loss of mitochondrial DNA under genotoxic stress conditions in the absence of the yeast DNA helicase Pif1p occurs independently of the DNA helicase Rrm3p. *Mol Genet Genomics* **281**, 635-45 (2009).
4. O'Rourke, T.W. et al. Differential involvement of the related DNA helicases Pif1p and Rrm3p in mtDNA point mutagenesis and stability. *Gene* **354**, 86-92 (2005).
5. Paeschke, K., Capra, J.A. & Zakian, V.A. DNA replication through G-quadruplex motifs is promoted by the Saccharomyces cerevisiae Pif1 DNA helicase. *Cell* **145**, 678-91 (2011).
6. Azvolinsky, A., Dunaway, S., Torres, J.Z., Bessler, J.B. & Zakian, V.A. The S. cerevisiae Rrm3p DNA helicase moves with the replication fork and affects replication of all yeast chromosomes. *Genes Dev* **20**, 3104-16 (2006).
7. Freudenreich, C.H., Kantrow, S.M. & Zakian, V.A. Expansion and length-dependent fragility of CTG repeats in yeast. *Science* **279**, 853-6 (1998).
